# Scale-up of the DMPA-SC in Nigeria: Why policy matters

**DOI:** 10.1186/s12905-022-02109-x

**Published:** 2022-12-21

**Authors:** Oluwaseun Akinyemi, Nicole Danfakha, Adewole Adefalu, Ebony Easley, Kayode Afolabi, Olajimi Latunji

**Affiliations:** 1 Department of Health Policy and Management, College of Medicine, University of Ibadan, Ibadan, Nigeria; 2 John Snow Inc., Capitol Heights, MD USA; 3 John Snow Inc., Abuja, Nigeria; 4 John Snow Inc., Philadelphia, PA USA; 5 Federal Ministry of Health, Abuja, Nigeria

**Keywords:** DMPA-SC, Sayana press, Family planning, Policy implementation, Scale-up

## Abstract

**Background:**

Injectable contraceptives have contributed substantially to Nigeria's rise in modern family planning methods usage. They are one of the most commonly used and preferred means of contraception among women in the country. Enabling policies are required to assure contraceptive access, security, and use. This study aimed to investigate the policy environment and how it supports or limits Nigeria's introduction and scale-up of subcutaneous depot-medroxyprogesterone acetate (DMPA-SC).

**Methods:**

The design of this mixed-methods study was cross-sectional. Desk reviews of policy papers, key informant interviews, and in-depth interviews were used to obtain information from respondents about the introduction of DMPA-SC in Nigeria and how existing policies influenced its scale-up. Data on DMPA-SC and other injectables were gathered from Nigeria's national electronic logistics management information system.

**Results:**

The findings suggest that policies such as task-shifting and task-sharing, cost-free policies, reproductive health policies, and others created an enabling environment for the scale-up of DMPA-SC adoption in Nigeria. The inclusion of DMPA-SC on the essential medicines list and the approved patent medicines list facilitated the scale-up process by ensuring private sector participation, removing economic barriers to access, fostering greater collaboration among health worker cadres, improving intersectoral partnerships, and improving logistics and client access. Despite significant anomalies in some implementing policies, injectable contraceptive consumption data demonstrate a progressive increase in DMPA-SC use during the study period. The results also indicate that policy initiatives have a favorable impact on the use of DMPA-SC throughout the country.

**Conclusion:**

The existence of policies, the active participation of stakeholders, and the political will of the Nigerian health system's leadership have all aided in the scaling-up of the DMPA-SC. Understanding how to build an enabling policy climate is critical for providing women with family planning options. These lessons from Nigeria emphasize the importance of these levers, which should be considered by teams intending to introduce innovative health products, particularly in developing countries.

**Supplementary Information:**

The online version contains supplementary material available at 10.1186/s12905-022-02109-x.

## Background

Only 19% of married women aged 15–49 in Nigeria utilize modern contraception, even though 98% of sexually active unmarried women and 94% of married women are aware of their options [1, 2]. In Nigeria, the public sector provides more modern contraceptive methods than the private sector (54% vs. 41%). Female sterilization (75%), intrauterine devices (79%), implants (93%), and injectables (74%) are provided mainly by the public sector [2]. However, male condoms (81%) and emergency contraception (80%) come from the private sector. Implants and injectables are usually purchased through government health centers (47%) and hospitals (41%). Patent medicine stores, also known as chemists, are the primary source of emergency contraception, male condoms, and pills [2, 3]. As of 2021, over 6 million women used a method of contraception. This led to the prevention of more than 2 million unintended pregnancies and the aversion of 756,000 unsafe abortions and 14,000 maternal deaths.

The uptake of family planning has remained low in Nigeria due to both client and health system challanges. A systematic review by Akamike and colleagues showed that the country's family planning utilization ranged from 10.3 to 66.8% [4]. Client-related challenges include lack of education, the desire for more children, uncertainty about the need, partner disapproval, previous side effects, religious beliefs, cultural disapproval, age, marital status, wealth index, place of residence, ignorance, humiliation, and domestic violence. At the same time, clients’ access to health services are impacted by limited access, including the cost of products and services, and procurement issues [4]. These challenges and obstacles to contraceptive uptake are more significant in Northern Nigeria for several reasons, including the patriarchal social structure, low education levels, and high poverty rates among women [5].

The Nigerian government works with key stakeholders to address large family preferences, religious precepts, and women's lack of sexual and reproductive health decision-making [6]. Nigeria, with a population of more than 200 million people, runs a federal system of 36 federating units called states, and a Federal Capital Territory, Abuja. Each state comprises Local Government Areas (LGAs) or counties [7]. There is a total of 774 LGAs in the country [7]. Health policies are usually made at the federal level and implemented at the state and LGA levels. However, states and LGAs have the power to make their health policies.

Nigeria plans to delegate its national family planning program to the states, improve access to services and commodities, and restrict population increase, leading to a healthier future for women and families [8]. Program priorities include dispelling family planning myths, expanding services and supplies, and enabling women and girls to make educated health decisions [6]. By 2030, Nigeria’s goals include ensuring that adolescents, youth, crisis-affected groups, and other vulnerable populations to make educated choices, have fair and affordable access to family planning, and equitably participate in society's development [6].

Subcutaneous presentation of lower-dose depot medroxyprogesterone acetate (DMPA-SC) is a new injectable administered under the skin. Sayana Press is a registered trademark of Pfizer Inc. Sayana Press, the DMPA-SC product available to Family Planning 2020 (FP2020) countries, is manufactured by Pfizer Inc. and combines the drug and needle in the prefilled BD Uniject™ injection system, which was initially developed by PATH (Program for Appropriate Technology in Health). The DMPA-SC in the Uniject™ is a 3-month contraceptive. It is one of the most convenient and effective family planning methods that may reduce some barriers to contraceptive access. DMPA-SC in Uniject can be administered in low-resource, non-clinic settings by a trained lower-level cadre of health workers or women themselves [9, 10]. Like in other Sub-Saharan African countries, injectable contraceptives are a prevalent method in Nigeria, accounting for 22% of the modern family planning method mix [9, 11]. In addition, there has been a rising trend in their popularity over the past 2 decades [12]. Convenience, affordability and improved privacy are some reported reasons for the use of injectable contraceptives [13]. These qualities were conferred by a conducive policy environment [14].

DMPA-SC was introduced through the private sector in Nigeria in January 2015 by DKT Nigeria, a nonprofit organization specializing in contraceptive social marketing [15]. In 2016, public sector delivery was launched and coordinated by UNFPA Nigeria and implemented through three local non-governmental organizations [NGOs] [16]. Nigeria leveraged existing programmatic materials, including PATH's DMPA-SC advocacy instruments and training materials which were adapted for Nigeria for service provision including counseling [15, 17]. Also, PATH was instrumental in developing the advocacy pack to facilitate the scale-up of DMPA-SC in Nigeria and worldwide [17].

An enabling policy environment is essential to successfully introducing and scaling health innovations [18, 19]. Policy formulation and implementation is an indispensable aspect of family planning programs as it ensures that all that is needed, for example, financial support, guidelines, training, regulations, and supplies, are in place to facilitate the uptake of contraceptive use, leading to increased contraceptive prevalence [20]. The availability of policies that improve contraceptive access has been shown to increase contraceptive use [21]. This has been true in Nigeria and other African countries, where for example, the community-based distribution policy was critical for the uptake of injectable contraceptives because it removed the access barrier for many people who could not go to health facilities for one reason or another [14]. Researchers have alluded to the need for policy frameworks to boost family planning use, identify common adverse reactions, and focus on educating the public about the negative implications of poor family planning uptake on people, families, and the country [1, 4]. Also, studies have shown that the lack of clear policy and implementation guidelines limited innovation within Nigeria's health system, including the introduction of new contraceptive technologies. This results in the deficiencies in funding, human resource for health planning, and logistics management during the new product scale-up process [14]. Therefore, policies must be implemented correctly for innovations to spread [22].

In Nigeria, several policies have been aimed at advancing the scale-up of DMPA-SC and self-injection in the country, for example, the task-shifting and task-sharing (TSTS) policy [9, 14]. The TSTS policy enabled injectable contraceptives to be provided in the community by community health extension workers (CHEWs). Thus, after a successful pilot, injectable contraceptives were provided nationally at the community level by CHEWS.

**Fig. 1:**
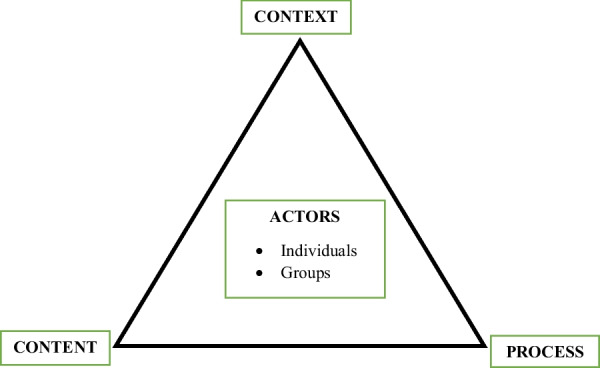
Health policy triangle framework (Adapted from Walt and Gilson [23])

This research draws on the policy triangle framework proposed by Walt and Gilson [23] (Fig. 1). This simple policy framework has four essential and interrelated components—context, content, process, and actors (individual and group stakeholders). Table 1 summarizes the relevance of each element in the framework to the appraisal of the scale-up of DMPA-SC in Nigeria.

**Table 1:** Applying the health policy triangle framework (Adapted from Walt and Gilson [23])

Steps in the framework	Application to the Scale-up of DMPA-SC in Nigeria
Context	What are the political, regulatory, cultural and socio-economic environment that influence the uptake of DMPA-SC in Nigeria?
Content	What are the sources and validity of data (primary and secondary) for this analysis? Is the policy evidence-informed?
Process	What are the activities that led to the formulation of the policy? Was there adequate statekeholder engagement?
Actors	Who are the drivers of the policy? Are they individuals, the state, development partners, NGOs?

The policy lessons learned from the scale-up of DMPA-SC in Nigeria may be applied to future policy analysis efforts and health innovation diffusion in the country and similar contexts. This study examined the influence of enabling policies in introducing and spreading DMPA-SC and self-injection in Nigeria to promote the country's family planning adoption.

## Methods

### Study design

This mixed-methods study was cross-sectional in design. The study is based on three datasets: a rapid literature review of policy documents, key informant, in-depth interviews (KII and IDIs), and family planning program data set from Nigeria's National Electronic Logistics Management Information System (eLMIS). Due to the restrictions associated with the COVID-19 pandemic, the interviews were all carried out over the phone.

### Participants and sample

Participants for the KIIs and IDIs were recruited through purposive sampling based on interest, availability, and ability to provide relevant information to the research question [24]. The number of interviews was based on the principle of saturation, where no new information was obtained from subsequent interviews [25, 26].

### Data collection

The first author (a lecturer and public health physician with a Ph.D., male, with about 10 years of experience in qualitative research), conducted the interviews and a research assistant (MPH, male, with 3 years of experience in qualitative research). Participants were first sent a mail to introduce the researchers (affiliations, professions, specialties, qualifications), the study, and its objectives, to seek their consent to participate in the research, record the interviews, and choose a conducive time for the interviews. Apart from the community resource person, interviews with other participants were conducted at the workplace. Only the participants and researchers were present at the interview. Here, we distinguish KIIs and IDIs as KIIs were done with policymakers and health system managers to generate information and ideas and explore the main issues surrounding DMPA-SC scale-up and the influence of policies in Nigeria [27]. The IDIs, however, pursue profound information and knowledge by exploring key themes that emerged from KIIs or have already been reported in the literature with health workers involved in the program's implementation [28]. Some of the themes explored include regulatory approval and its effect on scale-up, the influence of the accelerated introduction on the rapid uptake of DMPA-SC, the role of the task shifting/task sharing policy, and the introduction of self-injection on the diffusion of DMPA-SC. The IDI and KII guides used to lead the interviews were developed specifically for this study. The interview guides were pre-tested with individuals not participating in the study. The guides covered questions and prompt such as the appraisal of the policy environment for DMPA-SC scale-up, the role of stakeholders in the DMPA-SC scale-up in Nigeria, the perceived advantages and disadvantages of DMPA-SC as a form of the modern family planning method, and challenges with the introduction and scale-up of contraceptives, among others (Additional file 1).

KIIs were conducted with two senior managers at the Federal Ministry of Health and state/subnational managers at state ministries of health—one state per geopolitical zone. IDIs were conducted with healthcare providers, including patent and proprietary medicine vendors (PPMVs), community pharmacists (CPs), junior community health extension workers (JCHEWs), and community resource persons (CORPS). One category of providers in each selected state (two states well trained to be chosen purposively from JSI partners). IDIs were also conducted with two to three community gatekeepers per state. Transcripts were returned to interested participants to validate that they were genuine expressions of their views and for them to provide feedback on the study’s findings. All participants were at least 18 years old. Health workers included in the study had worked at least 6 months at the location.

The desk review entails a rapid review of policy documents—published and grey literature. The policy documents were primarily obtained from the Nigeria Federal Ministry of Health, some development partners, and the website of some organizations involved in family planning programming in Nigeria. The policy documents were examined under the lens of the policy triangle framework proposed by Walt and Gilson [23]. The broad questions the desk review seeks to answer are: What are the barriers and facilitators to the scale-up of DMPA-SC in Nigeria? In what way(s) did the policy framework provide a conducive environment for the uptake of DMPA-SC in Nigeria? Who were the actors in the policy process, and how was the policy developed? Some of the policy documents reviewed are summarized in Table 3.

### Data analysis

The interviews were recorded, transcribed, and analyzed using the thematic framework approach to qualitative data analysis using NVIVO (version 10). Notes were taken during the interviews to supplement the recordings. Also, three researchers independently coded and later compared and merged their codes to ensure inter-coder reliability [29]. In the course of the analysis, emerging themes were also added to already identified themes. Secondary data about DMPA-SC and other injectables’ consumption, triangulated from the National Electronic Logistic Management Information System (eLMIS), were used for the quantitative part of the study. The COREQ (COnsolidated criteria for REporting Qualitative research) Checklist was used in the preparation of this manuscript [30]. See Additional file 2.

Interviews were done over the phone, and the recordings and transcripts were produced. NVIVO version 10 and the thematic framework method for qualitative data analysis were employed in the study [31, 32]. Consumption data for DMPA-SC and other injectables were analyzed using Tableau, a public visualization tool, and presented using Microsoft Excel.

### DMPA-SC and other injectables consumption data

Nigeria’s consumption data for Depo-Provera, Noristerat, and subcutaneous depot medroxyprogesterone acetate (DMPA-SC, brand name Sayana® Press) from April 2018 to December 2020 were triangulated from the National Electronic Logistics Management Information System (eLMIS). An average of 10,884 facilities nationwide are reported within each bi-monthly period. Data from the eLMIS is uploaded into the Access Collaborative Dashboard. The following indicators were included in the analysis:

- Number of DMPA-SC units dispensed to clients by reporting period.
- Number of DMPA-IM units dispensed to clients by reporting period.
- Number of Noristerat units dispensed to clients by reporting period.
- The proportion of service delivery points (SDPs) that are stocked out of DMPA-SC.
- The proportion of service delivery points (SDPs) that are stocked out of DMPA-IM.
- The proportion of service delivery points (SDPs) that are stocked out of Noristerat.

## Results

As shown in Table 2, 19 participants were interviewed for the study between August and September 2020. There were no refusals, drops out, or repeat interviews. Each of the interviews lasted between 30 min and 1 h. More than half (68.4%) were females, and (68.4%) were aged 40 and up, with a mean age of 42.9 ± 7.9 years. About half (55.5%) were healthcare providers. In comparison, about 42% were chosen from family planning implementing partners who have supported the government's role in the DMPA-SC scale-up in Nigeria, and one participant (5.6%) was a community resource person. The healthcare providers have a work experience ranging from 9 to 23 years.

**Table 2:** Sociodemographic characteristics of participants (N = 19)

Variable	n (%)
*Gender*	
Female	13 (68.4)
Male	6 (31.6)
*Designation*	
Healthcare providers	10 (55.5)
Community resource person	1 (5.6)
FP implementing partners	8 (42.1)
*Type of interview*	
KII	9 (44.5)
IDI	10 (55.5)

Please see Table 3 for a comprehensive list of active policies affecting DMPA-SC and self-injection scale-up in Nigeria.

**Table 3:** Policies and activities aimed at advancing the scale-up of DMPA-SC accelerated introduction in the country

	Policy and activities	Startup-end date*	Year	Contribution to DMPA-SC scale-up
1	Approval and registration of DMPA-SC branded product by the National Agency for Food and Drug Administration and Control (NAFDAC) for provider administration		2011	This regulatory endorsement paved the way for the legal use of DMPA-SC
2	Approval of DMPA-SC updated label to include an indication for self-injection		2016	Provided necessary information for the implementation of self-injection
3	National DMPA-SC accelerated introduction and scale-up plan	Feb 2017–Dec 2017	2018	Provided modalities for coordinating the activities of various stakeholders in the process of introducing and scaling up DMPA-SC
4	National guidelines for the introduction and scale-up of DMPA-SC self-injection	Oct 2018–Jan 2019	2019	Serves as a guide for the implementation of the DMPA-SC self-injection intervention
5	Revision of task-shifting/task-sharing policy	May 2018–Mar 2019	2019	The TSTS policies allowed the expansion of provider cadre that can administer injectables, inclusive of DMPA-SC. It allowed pharmacists, proprietary patent medicine vendors PPMVs, as well as community-based distributors to administer DMPA-SC
6	Inclusion of DMPA-SC in the Essential Medicines List (EML)	Sept 2017–Sept 2019	2019	The EML is the list of priority medicines, considered to be most effective, procured by the country to meet the healthcare needs in a health system [53]. Inclusion of DMPA-SC in both the prescriptive medicine and Patent and proprietary medicine vendors (PPMV) lists of the document. This review was to allow DMPA-SC to be listed as an essential medicine so it can form part of the national procurement, and also allow dispensing in Nigeria
7	Inclusion of DMPA-SC in the Approved Patent Medicines List (APML)	July 2018–Dec 2019	2019	The APML, a list of medicines that licensed PPMVs are legally allowed to stock and dispense [54], gave policy backing for DMPA-SC to be stocked and dispensed by PPMVs
8	The Family Planning Costed Implementation Plan (CIP) 2019–2023 was created and it included DMPA-SC	Mar 2019–Dec 2020	2019	This is aimed at ensuring the integration of DMPA-SC into broader Family Planning programming/reducing siloed support

*These dates reflect when input into the policies was completed not the launch dates. In most cases, implementation of the policies had begun before they were launched

### Regulatory approval of DMPA-SC

The National Agency for Food, Drug Administration, and Control (NAFDAC) approved and registered the DMPA-SC branded product for provider administration in 2011. Then, in 2016, NAFDAC approved the DMPA-SC updated label, including the self-injection indication. The justification for this approval and registration was based on evidence of the safety and efficacy of pilot programs from other countries, among other things.

Justification for approval and registration was provided to NAFDAC based on evidence from all the pilots and even from other countries, you know, neighboring countries, those African countries and other countries in highlighting safety, efficacy, based on data from the studies carried out for similar processes in other countries. (Healthcare Provider)

Following the revision of the DMPA-SC label, the FMOH began incorporating DMPA-SC self-injection into the country's introduction and scale-up plan. The scale-up plan, a five-year roadmap, lays the groundwork for the long-term integration of the DMPA-SC into Nigeria's healthcare cocktail of family planning methods [9].

### National DMPA-SC accelerated introduction and scale-up plan

The *National DMPA-SC Accelerated Introduction and Scale-up Plan* was introduced in 2018. The scale-up plan was developed in response to DKT and UFNPA pilots of DMPA-SC in southwest and southeast Nigeria in 2015 and 2016, documenting critical challenges in implementing DMPA-SC in Nigeria. The respondents confirmed that the plan was created to serve as a road map for implementing DMPA-SC and to aid in its adoption. Other responses are listed below:

You know it facilitates the uptake of DMPA-SC by considering some key implementation challenges, and even solutions to address them. (Healthcare Provider)

Well, the gap is, for me, two things. First of all, set a roadmap because this is a new intervention. This is a new product. That is the DMPA-SC. So, without a policy guiding how this can be ruled out, then it’s almost difficult for the state to be able to implement that intervention. (Healthcare Provider)

This policy update was timed to coincide with the conclusion of pilots assessing feasibility, allowing it to serve as a context-informed roadmap.

### Revision of the task-shifting/task-sharing policy

In 2019, the task-shifting/task-sharing policy was revised. This policy allows lower-cadre health workers, such as CHEWs, to perform tasks previously reserved only for doctors and nurses. This revision was carried out in response to the difficulties encountered regarding the healthcare worker cadre permitted to administer DMPA-SC, according to the responses from the interviews. Some of the answers are provided below in the form of quotes:

There were difficulties [previously] in terms of which health worker cadre could provide it, but subsequently, the task-shifting task sharing policy, CHEWs, and even JCHEWs were permitted to give injectable contraceptives. (FP Implementing Partner)

The task shifting and the task sharing policies that allow the lower cadre of providers like the community health officers, the CHEWS, and then also the PPMVs to be able to provide injectable contraceptives including the DMPA-SC is one policy that has also helped to facilitate the accelerated use of DMPA-SC. (Healthcare Provider)

The revision allowed more cadres of health workers to administer DMPA-SC, facilitating its uptake.

The thing that is very, very clear is that the SOP captures PPMVs and community pharmacists to provide DMPA-SC, you know, but the policy document itself does not because, at the time, we were revising, reviewing it, the sheer accreditation had not been approved by the President at that time. It was still awaiting presidential assent. And I think we're no longer following up on that. But that is a gap. (Healthcare Provider)

The revision of the task-shifting/task-sharing policy emphasizes the importance of supporting documents. While the National DMPA-SC Accelerated Introduction and scale-up plan outlined scale-up processes, it could not be fully implemented without the TSTS policy authorizing DMPA-SC administration by the cadres included in the scale-up plan.

### National guidelines for the introduction and scale-up of DMPA-SC self-injection

In 2019, the National guidelines for introducing and scaling up DMPA-SC self-injection were completed. The policies were deemed vital because they provided additional detail to the initial policy in order to guide program implementation, including instructions to ensure that self-administration was done safely and correctly. According to the health managers:

So why that guideline is so crucial and adherence to it is very important is because you want to be sure that this woman administers DMPA-SC to herself safely, can follow the instruction, can calculate the next date for the following dose, and also can keep the used unit safely until she’s returning to the facility when she will now have to hand it over to the provider who will check that the used unit is complete. (FP Implementing Partner)

Well, it did focus a little bit. It mentioned a few things about self-injection…most of it is health facility-focused. It is around the health worker rather than more of how do we empower women to give themselves the injection. (FP Implementing Partner)

The national guidelines for the initiation and scaling-up of DMPA-SC self-injection are essential because they provide comprehensive guidance and a detail a woman's journey through self-injection from initiation to administration and follow-up. Following the policy's official launch in April 2019, there was a rapid increase in partner rollout of DMPA-SC training, possibly because partners were now more confident in the beginning full implementation of self-injection as part of their FP training. In addition, there was a noticeable increase in the proportion of DMPA-SC in the injectable method mix from 6 to 11% (before and after the policy's implementation) and a subsequent trend of continuous increase [33].

### Inclusion of the DMPA-SC in the essential medicines list and approved patent medicines list

In 2019, DMPA-SC was added to the essential medicines list (EML) and the approved patent medicines list (APML). According to respondents, this inclusion increased the country's contraceptive method mix and helped to ensure that the product was easily accessible in both public and private facilities:

Okay, so for inclusion in the EML and APML, of course, it improved the method mix, thereby increasing users’ choices. This assured procurement (guaranteed provision) of the commodity because the government buys what is in the EML and then distributes it in batches to enable a wider range of providers. Consequently, still expanding the market and reach to communities, especially hard-to-reach communities. (Healthcare Provider)

The inclusion of DMPA-SC also reduced the level of stock out rate through the fact that when there is stock out in the facilities, for example, women can have access to go to the private facility to ask that. And then, it aided the last mile for commodity distribution, and an emergency of focal commodities can be procured from government-approved facilities within the country. (FP Implementing Partner)

Without DMPA-SC being on the EML list and as an over-the-counter product, it means that the PPMVs won’t be able to stock it, which means refill will be a challenge at the community level. So that’s the major thing there. (FP Implementing Partner)

The inclusion of DMPA-SC in the EML and APML, ensured the availability of the product by enabling government and private providers to procure and stock the product. This was a critical step in supporting scale-up.

### DMPA-SC and family planning costed implementation plan

In 2019, the Family planning costed implementation plan (CIP) 2019–2023 was created, which included DMPA-SC. The inclusion of DMPA-SC ensured that the product would be included in the basket of commodities to be procured and managed by government as well as in programming efforts, including demand generation activities. after 2019, DMPA-SC was included as part of national procurement exercises.

It ensures that on the supply side, we make provision for DMPA-SC forecast, quantification, and procurement planning. Of course, without the commodity available, you can do nothing about it. So that was the key thing on the supply side. Then the other thematic area in the blueprint… was demand generation. Demand generation effort in the country, we have to incorporate DMPA-SC into it. So, all our demand generation activities are now incorporated with DMPA-SC and self-injection. So all these were contributions from the CIP. (FP Implementing Partner)

### Perceived success levers for scale-up

When asked what they believe are the critical success levers for the scale-up of DMPA-SC, some respondents mentioned the Federal Government's collaboration with other implementing partners in developing policies and guidelines. The guidelines were developed using a bottom up approach, meaning that stakeholders from the sub-national levels were engaged in the policy development process. Effective policy change was achieved through the engagement of leadership at all levels of the health system in the process.

Okay, so, for me, I think the key lever is government buy-in and buy-in of stakeholders. If everybody is on the same page, that's one. Secondly is the availability of evidence as well. You understand. These are the two key levers that come to mind now. (Doctor, NGO)

So awareness is quite critical even among us policymakers as well as program managers and even service providers. Then articulating policies and guidelines to create enabling environment to be all-inclusive. The bottom-up approach has been very effective because of ownership. (Senior Health Manager, FMOH)

### Consumption and stockout data for DMPA-SC and other injectables

**Fig. 2:**
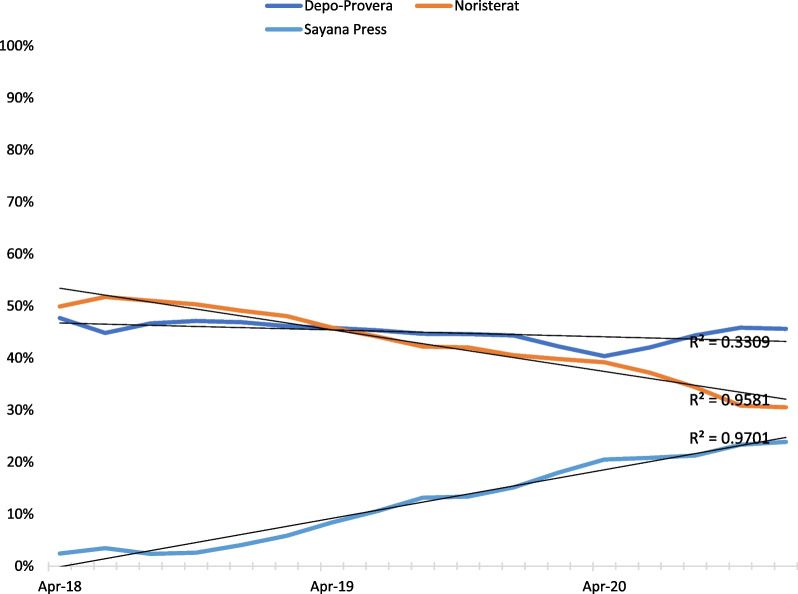
Injectables consumption over time

Figure 2 shows that while the uptake of other injectables appears to be declining (Noristerat) or plateauing (Depo-Provera), the uptake of DMPA-SC (Sayana Press) gradually increases during the study period. The rise in DMPA-SC consumption was accompanied by a strong linear trend (R^2^ = 0.97). The increase observed from early to late 2019 appears to correspond with policy interventions–revision of the TSTS policy and the inclusion of DMPA-SC in the EML and APML (all in 2019).

**Fig. 3:**
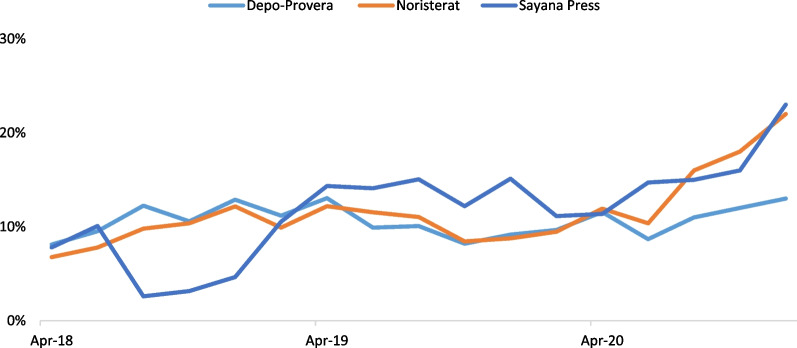
Stockout of injectables over time

Figure 3 depicts injectable stockout data from April 2018 to December 2020. According to available data, DMPA-SC stockout was higher than other injectables in 2019, and stockout for all injectables generally spiked in the second half of 2020. According to the two-tailed, paired T-test, there was no statistically significant difference in stock-out rates between Sayana Press and Depo Provera (*p* = 0.38) or Sayana Press and Noristerat (*p* = 0.81).

## Discussion

In this study, we collected data on how specific policies influenced the introduction of DMPA-SC in Nigeria. This section discusses the implications of the findings in promoting the country’s family planning uptake.

### Role of evidence in the policy process

Respondents in this study stated that the DMPA-SC was implemented based on the best available evidence, both internationally and locally. According to researchers, successful policies are evidence-based, specific, and have explicit goals [34, 35]. Baicker and Chandra [34] posited that evidence-based health policy is critical for making rational policy decisions and for focusing policymakers' and political leaders' attention on health innovations that have the potential to improve people's lives. In addition to evidence, context is critical for successful policy implementation because it influences how evidence is interpreted and utilized. [36]. As a result, Nigeria needed to conduct its pilot study on CBD injectable contraceptives by community health workers rather than relying solely on evidence from other African countries before proceeding [37]. Furthermore, research has shown that implementing health innovations is context-specific, particularly in low- and middle-income countries (LMICs) with varying health system capability [14]. As a result, policy implementation that takes evidence, context, and timeliness into account can withstand the test of time and be replicated in other contexts [34, 36]. However, 'evidence' in this situation combines traditional academic and political evidence—efficiently combining these can often be challenging [38].

### Imperative of a conducive policy environment for DMPA-SC implementation and scale-up

Furthermore, the findings of this study revealed that the National DMPA-SC Accelerated Introduction and Scale-up Plan, as well as the task-shifting/task-sharing policy revision, created a favorable environment for the DMPA-SC's implementation in the country. According to research, the availability of contextual policies aids in better engaging stakeholders in providing equitable health services, thereby closing the gap in the delivery of health services, including the provision of DMPA-SC, to those living in poverty [39]. Moreover, the lack of a favorable policy environment has been identified as a significant reason why some proven practical innovations are not successfully scaled up in LMICs [14, 40].

Furthermore, participants in this study stressed the significance of national guidelines for introducing and scaling up DMPA-SC self-injection in safely providing DMPA-SC to interested clients. The guideline provides the necessary information for the implementation and uptake of a self-injection plan by covering most issues from policy, advocacy, logistics, client and provider training, demand generation, and monitoring. In a study done in Malawi, Burke and colleagues emphasized the importance of implementation guidelines in the process of DMPA-SC scale-up [41].

### Implications of policy inconsistencies in the scale-up process

Despite those above, a critical examination of some policy documents reveals contradictory instructions and a lack of clarity in the guidelines. For example, the TSTS Standard Operating Procedure [10] states that PPMVs and other community-based health workers can "counsel, initiate, and maintain injectable contraceptives.” In contrast, the Self-injection guidelines [42] outline that PPMVs are only allowed to refill, follow-up, counsel, mobilize the community, and refer. This contradicts an essential feature of effective policy, as previously described—specificity, consistency, and clarity of goals. The consequence of ambiguity on policy impact has been documented in the literature [43–46]. It may also exacerbate role conflicts [47] and inter-cadre rivalry, which has historically negatively affected the Nigerian health system [14, 48].

According to the results of this study, the inclusion of the DMPA-SC in the essential medicines list (EML) and the approved patent medicines list (APML) in 2019 contributed to women’s choices by broadening the contraceptive method mix. When national procurement of DMPA-SC begins, the inclusion will ensure long-term access to the commodity by providing availability from private (patronized by approximately 60% of patients) and government-owned health facilities [49]. Furthermore, including the DMPA-SC in both the EML and the APML ensures that private providers can provide injectable contraceptives [49].

The family planning costed implementation plan (CIP), as demonstrated by the findings of this study, ensures a balance between the demand for and supply of contraceptive commodities (including the DMPA-SC) and everything in between. In Nigeria, the CIP serves as a multiyear coordinated plan to achieve government goals and assists in allocating limited resources to the products and methods that will significantly impact achieving these goals. According to the Health Policy Project [50], CIPs are critical frameworks that assist governments in achieving their family planning objectives effectively and efficiently. CIPs are detailed plans that cover various family planning thematic areas such as demand generation, contraceptive security, service delivery, access, financing, stewardship, and accountability [50, 51]. As a result, CIPs are critical to the successful implementation and scale-up of health innovations such as the DMPA-SC.

### Limitations

Like any cross-sectional study, this study does not imply any causality between the described levers and effective policy implementation. The study does not explore the level of effort to advocate for and craft policies since its focus is on scale-up with current policies already in place. Furthermore, even though respondents were assured of complete confidentiality, social desirability and recall bias are possible. Also, reports from similar contexts revealed that there was no reporting on actual consumption from service delivery points, little or no data visibility at the central level or among decision-makers, and no LMIS standard operating procedures [52]. This may also be true of data from Nigeria’s eLMIS used in this study. However, this study provides valuable lessons for policymakers and public health program implementers, particularly in LMICs.

## Conclusion

Policies must be put in place to allow for the introduction of new health products. These policies should be evidence-based, detailed, consistent, and have defined objectives. Effective policies should be contextual, timely, and involve stakeholders at all levels of the health system across the private and public sectors, in addition to being evidence-based. The scale-up of the DMPA-SC has been aided by policies in place, the active participation of stakeholders, and the political will of the Nigerian health system's leadership. Furthermore, client-centered innovations, such as self-injection, aided in contraceptive usage. In conjunction with stakeholders, the health leadership's policies and efforts created an enabling environment for the introduction and scale-up of the DMPA-SC, albeit some inconsistencies in the policies make implementation potentially chaotic. Establishing an enabling policy climate is critical as we strive to empower clients with family planning options. These lessons from Nigeria emphasize the importance of these levers, which should be considered by teams intending to implement innovative health products, particularly in low- and middle-income countries.

## Supplementary Information

**Additional file 1.** Interview guide.

**Additional file 2.** COREQ (COnsolidated criteria for REporting Qualitative research) Checklist.

## Data Availability

Data for this study are available upon request. Datasets used during the current study is available from the corresponding author on reasonable request.

## Abbreviations

APML: Approved patent medicines list
CORPS: Community resource persons
CP: Community pharmacist
DMPA-SC: Subcutaneous depot-medroxyprogesterone acetate
DMPA-IM: Intramuscular depot-medroxyprogesterone acetate
eLMIS: Electronic logistic management information system
EML: Essential medicines list
FP: Family planning
IDI: In-depth interview
JCHEW: Junior community health extension worker
JSI: John Snow Inc.
KII: Key informant interview
NGO: Non-governmental organization
PATH: Program for appropriate technology in health
PPMV: Patent and proprietary medicine vendors
SDP: Service delivery point
TSTS: Task-shifting and task-sharing
UNFPA: United Nations Population Fund
