# Supplementary material for: Scale-up of the DMPA-SC in Nigeria: Why policy matters
Source: BMC Womens Health. 2022 Dec 21;22:535. doi: 10.1186/s12905-022-02109-x (PMC9768394; doi:10.1186/s12905-022-02109-x)
Supplement: Supplementary file 1 — Additional file 1. Interview guide. [file 12905_2022_2109_MOESM1_ESM.docx]

**Key Informant Interview Guide**

KII Guide for:

- **Senior managers at Federal Ministry of Health (FMOH)**
- **State/Subnational managers at State Ministry of Health (SMOH)**
- **Partners**

Introduction: Good morning/afternoon Sir/Ma. Thank you so much for honoring our invite and agreeing to talk with us today. My name is……………... We are trying to gather information on the ***facilitators and barriers to the scale-up of the DMPA-SC in Nigeria***.

1. What is your role in the scale-up of DMPA-SC in Nigeria?
2. Can you please tell us the status of DMPA-SC as a form of contraception in Nigeria?
3. Are there any advantages or disadvantages of DMPA-SC as a form of contraception in Nigeria?
4. Have you had challenges around availability of DMPA-SC?
5. How has COVID 19 pandemic affect availability and accessibility to DMPA-SC?
6. How have you been coping with stock out?
7. Can you briefly work us through the challenges around introducing/scaling-up of DMPA-SC in Nigeria?
8. What are the factors that have helped in the scale up of DMPA-SC in Nigeria?
9. In what areas of the DMPA-SC introduction do you think we need more research?

Probe: What type of research do you think is needed specifically?

1. In your opinion, what policies have contributed the most to the introduction and scale up of the DMPA-SC in Nigeria?

Probe:

- What is the role of the TSTS policy in DMPA-SC introduction in Nigeria?
- Who are the critical stakeholders in the implementation of these policies? Do you think they were involved in the formulation & implementation processes?
- What other policies do you think are necessary for more effective scale-up of the DMPA-SC in Nigeria?

1. How have these policies influenced the country’s health systems?

Probe: Consider the health systems building blocks – Leadership/governance, health care financing, health workforce, medical products/technologies, information & research and service delivery

1. Are there any additional suggestions/ideas you would like to share that we have not talked about?

Thank you very much for participating in this study.

**In-depth Interview Questions**

IDI Guide for:

- **Healthcare workers**

We are trying to gather information on the ***facilitators and barriers to the scale-up of the DMPA-SC in Nigeria***.

1. What is the status of DMPA-SC as a form of contraception in your facility/community?

Probe:

• What is your role in the distribution of the DMPA-SC in the facility/community?

2. Please describe any government policies related to DMPA-SC which you are familiar with?

Probe: What are they? Do you think these policies facilitated women using DMPA-SC?

3. What is your opinion on DMPA-SC and SI as a contraceptive option for the country? Are women ready for this? Is the country ready for this?

Probe: From your experience, how well do you think women handle the self-injection of this contraceptive?

4. Can you describe how you talk to women about DMPA-SC?

5. How would you describe the women who are most interested in DMPA-SC/SI?

a. Age, wealth status, education, marital status etc.

- 1. b. Are women switching from other form of contraception to DMPA-SC?

6. What are some factors that interest clients about DMPA-SC?

Probe:

• What is your opinion of DMPA-SC? Does it add value to the FP program? Are there challenges around introducing DMPA-SC to women coming to your facility?

• What about the factors that have assisted in introducing DMPA-SC to women coming to your facility?

• What are the barriers to the uptake of DMPA-SC by women in your facility/community?

7. Have women had any issue with side effect of DMPA-SC?

Probe: What are these side effects?

8. What challenges have you encountered in administering DMPA-SC?

9. Have you had challenges around availability of DMPA-SC?

Probe:

• How has COVID 19 pandemic affect availability and accessibility to DMPA-SC?

• How have you been coping with stock outs?

10. What can or should the government do to support making DMPA-SC available to women as a contraceptive option?

11. Are there any additional suggestions/ideas you would like to share that we have not talked about?

Thank you very much for your time.

**In-depth Interview Guide**

IDI Guide for:

- **Community Gate Keepers**

Introduction: Good morning/afternoon Sir/Ma. Thank you so much for honoring our invite and agreeing to talk with us today. My name is……………... We are trying to gather information about Subcutaneous Depo Medroxylprogesterone Acetate (DMPA-SC) and we are interested in finding out what you know about DMPA-SC among women. The information you will be sharing will be confidential. The information discussed today will assist us to have a better understanding and to scale-up DMPA-SC in Nigeria. We would like to record this conversation and all the recording will be kept safe. Your participation is completely anonymous. If at any moment you do not want to proceed with this interview, you are free to discontinue. This interview will last about 30 – 40 minutes. Thank you once again.

1. Please can you tell us your position in the community?
2. What are the types of family planning methods that you know? *If DMPA-SC is not mentioned, ask the next question*
3. Have you heard of DMPA-SC?
4. What is your opinion about DMPA-SC as form of modern family method for the women in your community?

Probe: What are the advantages of the DMPA-SC over other types of contraceptives?

1. Have you heard of any side effect of DMPA-SC from the women in your community?
2. In your opinion, what do you suggest or what can be done to make women have greater access to DMPA-SC in your community?

Probe: Are there policies you would like to see from government to improve women’s access to DMPA-SC?

1. Are there any additional information you would like to share that we have not talked about?

Thank you very much for your time.
